# Bibliographical Notices

**Published:** 1842-04-09

**Authors:** 


*An Historical Sketch of the State of American Medicine before the Revolution.* By John B. Beck, M. D., Professor of Materia Medica and Medical Jurisprudence in the College of Physicians and Surgeons of New York. 8vo., pp. 35.

This pamphlet, the annual address for 1842, before the Medical Society of the State of N. Y., is one of many excellent contributions to our medical literature, for which we are indebted to the industry and talent of Prof. J. B. Beck. The materials from which this sketch is worked up have been collected from a great number of different sources, and the author is entitled to the particular thanks of the profession for the time and labour expended upon a work which must be read with universal interest. We hope it may be generally circulated, for we are sure that every American physician will be glad to peruse it. The following extract, which contains an account of our Philadelphia ante-revolutionary physicians, we select as a specimen of Dr. Beck's style:

"Among the medical men of Pennsylvania, there are several who are entitled to notice, as having contributed to the colonial literature of our profession. In 1740 Dr. Thomas Cadwallader, of Philadelphia, published 'An Essay on the Iliac Passion,' in which he exposes the absurdity of the practice then in vogue, viz: that of treating it by quicksilver and drastic purges. He recommends in their stead, mild cathartics, with the occasional use of opiates. By Dr. Thomas Bond, an eminent physician of Philadelphia, two communications were published in the London Medical Observations and Inquiries, one an account of a worm bred in the liver, 1754; another on the use of bark, in scrofulous cases, 1759. The men, however, who were particularly distinguished, in Philadelphia, for their zeal in the cause of medical science, were Drs. John Morgan and William Shippen, both natives of that place, and the founders of the first medical school established in this country.

Dr. Morgan, after studying medicine at home, went to Edinburgh, where he received the doctor's degree, on which occasion he published an elaborate thesis on the formation of pus—'Tentamen Medicum de Puris Confectione, Edinburgh, 1763.' In this dissertation he maintained the doctrine that pus is a secretion, prepared by a peculiar action of the secretory vessels of the part. The credit of originality, as it regards this doctrine, has generally been awarded to the celebrated John Hunter. The evidence, however, appears to be conclusive, that he was anticipated by Dr. Morgan. After receiving his degree at Edinburgh, he travelled for some time on the continent, industriously engaged in acquiring knowledge, and every where received with the highest honor. As a proof of the estimation in which he was held abroad, it is only necessary to state, that on his return home, in 1765, he was a fellow of the Royal Society of London, corresponding member of the Royal Academy of Surgery of Paris, and licentiate of the Royal Colleges of Physicians of London and Edinburgh. Notwithstanding his devotion to science, Dr. Morgan was not a prolific author. Besides his Thesis, all that we have left is his 'Discourse,' already noticed, 'On the Institution of Medical Schools in America,' in 1765, and 'A Recommendation of Inoculation, according to Baron Dimsdale's Method,' 1776.

Dr. Shippen, was born in 1736, and about the year 1760 took his degree at Edinburgh, on which occasion he wrote and published a thesis, 'De Placentæ Cum Utero Nexu." Besides this I do not know that he published any thing, but he is greatly and justly celebrated as the first person who lectured on anatomy in this country."

*On Regimen and Longevity, comprising Materia Alimentaria, National Dietetic Usages, and the Influence of Civilization on Health and the Duration of Life.* By John Bell, M. D., Lecturer on Materia Medica, &c. &c. Philadelphia, Haswell & Johnson, 1 vol. 8vo., pp. 420.

This book, although designed chiefly for the general reader, has claims upon the notice of the physician. It is the result of extensive reading, considerable experience, and much reflection upon the subjects treated of, and may be safely recommended by the profession to general perusal. It is written in a very agreeable style, interspersed with national and historical sketches and occasional anecdote, and is replete with sound opinions and valuable information. Dr. Bell has long enjoyed a high reputation as a writer on hygiene and kindred topics, to which his present excellent and elaborate production is fully equal. Our limits do not allow an extended analysis of a work of this character, and we must confine ourselves to the simple expression of approbation of the views and principles laid down by the author. On the subject of alcoholic drinks we think him slightly tinged with ultraism, and he strikes us as arguing the question, although ably, with more warmth than philosophy. It may be, however, urged, that in combating so wide spread an evil as intemperance, this extreme mode of presenting the other side of the question is allowable, and we are not prepared to gainsay the plea.

---

*Physiology for Schools.* By Reynell Coates, M. D., Corresponding Member of the National Institution, Washington City—and the New York Lyceum of Natural History; member of the Academy of Natural Sciences of Philadelphia, etc. etc. Second Edition, revised. Philadelphia: E. H. Butler. 1842.

This little volume is designed for the use of schools in which the plan of education is rather more extended than the usual field. We can only refer our readers to the volume itself, which is believed to include in a compact and agreeable plan the physiological information suitable for the class of readers for whom it is designed.

---

*Dictionnaire de Medécine, &c. Deuxième edition. Tome vingt-quatrieme.* Paris, 1841.

*Dictionary of Medicine, &c. Second edition. Twenty-fourth volume.* Paris, 1841.

The twenty-fourth volume of this dictionary brings the work to the letters P L A. In the present volume we find the important articles, Phthisis, Phlegmasia Dolens, Physiology, the Anatomy and Diseases of the Feet, Plague, &c. The work is not accessible to all our readers, but is indispensable to those who have frequent occasion to refer to different subjects connected with medical literature. The copious bibliographical references contribute very much to the convenience of the reader. The sale of this dictionary is much more extensive than is generally known. A number of copies are received in Philadelphia, for city and distant subscribers, much greater than of any other large work in a foreign language.

The article on phthisis, by Dr. Louis, is almost a condensed edition of his volume published in 1826, with the additional matter which he has since collected. It forms one of the most valuable monographs on the disease.

The surgical articles upon the feet and their diseases, are not inferior to the memoir of Dr. Louis in their practical value.

For the information of distant readers, we may state that the price of this work, when complete, will be about fifty dollars, at the present rate of duties.

